# Trends in incidence of self-harm, neurodevelopmental and mental health conditions among university students compared to the general population: a nationwide electronic data linkage study in Wales.

**DOI:** 10.23889/ijpds.v9i5.2535

**Published:** 2024-09-18

**Authors:** Olivier Rouquette, Sze Chim Lee, Jo Smith, Marcos del Pozo Banos, Ann John

**Affiliations:** 1 Swansea University Medical School; 2 University of Worcester

## Objective and Approach

They are growing concerns that self-harm and mental health conditions are increasing in university students. This may reflect widening access to higher education, existing population trends or stressors associated with this setting. We linked real-world data from the Higher Education Statistics Agency between 2012-2018 with primary and secondary healthcare records. Students were undergraduates aged 18 to 24 years at university entry. Non-students were pseudo-randomly selected based on an equivalent age distribution. We used regression approach to examine characteristics of students and non-students and their risks of self-harm and mental health conditions before and during university.

## Results

This e-cohort study included 96,760 students and 151,795 non-students. Being male, self-harm and mental-health conditions recorded before university entry, and higher deprivation levels resulted in lower odds of entering university and higher odds of dropout. Risks of self-harm, Autistic Spectrum Disorder (ASD), depression, anxiety, drug misuse, and schizophrenia were lower for students. Risks of self-harm, ASD, ADHD, depression, alcohol misuse and schizophrenia were increasing more in students over time. Older students experienced higher risk of self-harm and mental-health conditions. Younger students were more at risk of alcohol misuse than non-students.

## Conclusions

Mental health conditions in students are common, diverse and they influence their academic outcomes. University students have mental health needs and trajectories that differ from those of the general population.

## Implications

The diversity and complexity of students’ needs require integrated person-centred stepped care approach within universities including triage, access to well-being support, counselling, and referral pathways to mental health services..

